# Engineering NOG-pathway in *Escherichia coli* for poly-(3-hydroxybutyrate) production from low cost carbon sources

**DOI:** 10.1080/21655979.2018.1467652

**Published:** 2018-04-24

**Authors:** Yangyang Zheng, Qianqian Yuan, Hao Luo, Xue Yang, Hongwu Ma

**Affiliations:** a Key Laboratory of Systems Microbial Biotechnology, Tianjin Institute of Industrial Biotechnology, Chinese Academy of Sciences, Tianjin, China; b University of Chinese Academy of Sciences, Beijing, China

**Keywords:** genome-scale metabolic network analysis, *Escherichia coli*, glycerol, NOG pathway, Poly-(3-hydroxybutyrate), xylose

## Abstract

Poly-(3-hydroxybutyrate) (P3HB) is a polyester with biodegradable and biocompatible characteristics suitable for bio-plastics and bio-medical use. In order to reduce the raw material cost, cheaper carbon sources such as xylose and glycerol were evaluated for P3HB production. We first conducted genome-scale metabolic network analysis to find the optimal pathways for P3HB production using xylose or glycerol respectively as the sole carbon sources. The results indicated that the non-oxidative glycolysis (NOG) pathway is important to improve the product yields. We then engineered this pathway into *E. coli* by introducing foreign phophoketolase enzymes. The results showed that the carbon yield improved from 0.19 to 0.24 for xylose and from 0.30 to 0.43 for glycerol. This further proved that the introduction of NOG pathway can be used as a general strategy to improve P3HB production.

## Introduction

In our recent work, based on genome-scale metabolic network analysis, we designed an optimal pathway for P3HB production from glucose using the non-oxidative glycolysis (NOG) pathway and the construction of this pathway in *Escherichia coli* greatly improved P3HB product yield [1]. Considering that the cost of carbon sources is the most important factor affecting P3HB production cost [2], it is desirable to explore the possibility of using low cost carbon sources such as hemicellulose and glycerol for P3HB industrial production.

Hemicellulose is the third most abundant polymer in nature and can be easily hydrolyzed into fermentable sugars [3]. Xylose is the dominant building unit of hemicellulose. It has been reported that *Ralstonia eutropha* using xylose as a sole carbon source can produce 2.31 g/L of P3HB with a content of 30.95 wt% [4].

Glycerol is the main by-product of the biodiesel production. During the process of transesterification of vegetable oil with methanol or ethanol, 1 ton of glycerol is inevitably produced for every 10 tons of biodiesel [5]. Biodiesel-derived glycerol has potential to be used as a platform molecule for manufacturing commercially valuable chemicals [6]. It has been reported that engineered *E. coli* containing P3HB synthesis pathway could produce 8 g/L P3HB (65% content) in a 7.5 L bioreactor using crude glycerol as the sole carbon source [7].

In this paper, we first theoretically evaluate the effect of NOG pathway on P3HB production from xylose and glycerol through genome-scale metabolic network analysis. Based on the computational analysis results, we engineered *E. coli* strains to produce P3HB from xylose or glycerol and the higher P3HB yield in the strains with NOG pathway validated the theoretical prediction.

## Results

**Figure 1.**
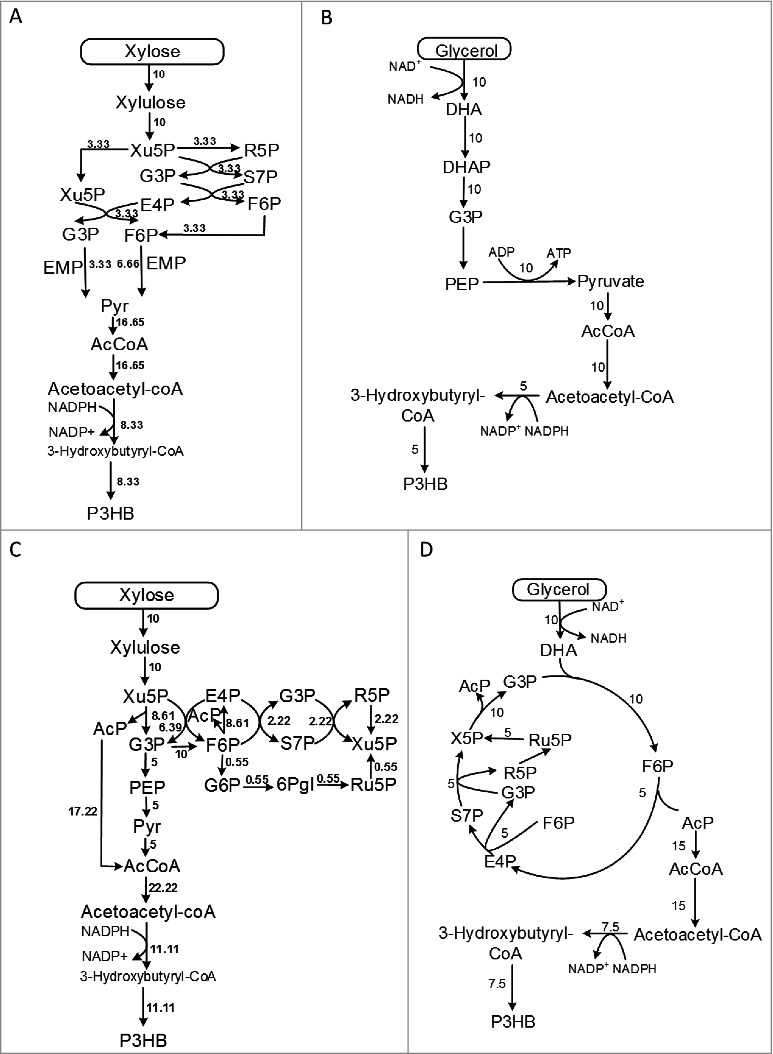
The original P3HB synthesis pathway (A, B) and the calculated optimal P3HB synthesis pathway ( C, D) with flux distribution in *E.coli* using xylose or glycerol as sole carbon source. The numbers show the reaction fluxes of each reaction. Abbreviations:Xu5P, xylulose 5-phosphate; R5P, ribulose 5-phosphate; G3P, glyceraldehyde 3-phosphate; S7P, sedoheptulose 7-phosphate; E4P, erythrose 4-phosphate; F6P, fructose 6-phosphate; Pyr, pyruvate; AcP, acetyl phosphate; DHA, dihydroxyacetone; DHAP, dihydroxyacetone phosphate; PEP, phosphoenolpyruvate.

1 Computational simulation indicates improved P3HB yield through the NOG pathway In our previous study, we showed that the introduction of NOG pathway in *E. coli* could increase the theoretical carbon yield of P3HB to glucose from 66.7% to 88.9% by calculating the optimal pathways for P3HB production using flux balance analysis of an extended *E. coli* metabolic network model [1]. The normal pathways for P3HB production from xylose and glycerol are shown in Fig. 1A and Fig. 1B respectively. Assuming the carbon source consumption rate was 10 mmol gDCW^−1^ h^−1^, the maximal P3HB monomer production rate was 8.33 mmol gDCW^−1^ h^−1^ and 5 mmol gDCW^−1^ h^−1^ respectively, equaling to a 66.7% carbon yield considering the different numbers of carbon element in the two substrates (5 in xylose and 3 in glycerol). The carbon loss is due to the release of CO_2_ in the pyruvate decarboxylation step. In contrast, the P3HB carbon yields of the optimal P3HB production pathways calculated from the extended metabolic network with NOG pathway was 88.9% for xylose (11.1 mmol gDCW^−1^ h^−1^ P3HB from 10 mmol gDCW^−1^ h^−1^ xylose) and 100% for glycerol (7.5 mmol gDCW^−1^ h^−1^ P3HB from 10 mmol gDCW^−1^ h^−1^ glycerol) as shown in Fig. 1C and 1D. These theoretical analysis results indicated that the introduction of NOG pathway was effective to improve the theoretical carbon yield for both xylose and glycerol.

2 Improved PHB/xylose yield through the introduction of NOG pathway into *E.coli*

**Figure 2.**
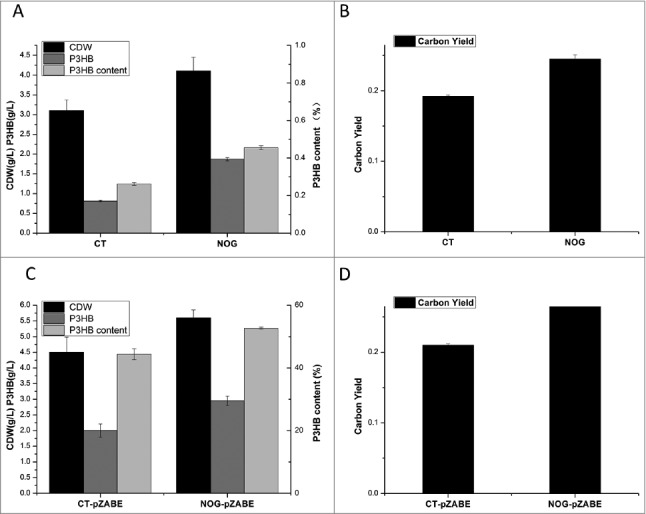
Comparison of CDW, P3HB concentration, P3HB content and the carbon yield using xylose as the sole carbon source (A,B) and using both glucose and xylose (C, D) in the control and engineered *E. coli* strains.

To verify the effect of NOG pathway on the P3HB synthesis using xylose as sole carbon source in MM medium [8], we introduced the NOG-pathway in *E.coli* by the over-expression of phosphoketolase (encoded by *fxpk*) and fructose-1,6-bisphosphatase (encoded by *fbp*) [1]. Shake flask experiments were conducted using 15 g/L xylose as the sole carbon source. Cells were harvested after shaking at 220 rpm and 37 °C for 48 h. The results of the cell dry weight, P3HB weight, P3HB content and carbon yield were shown in Fig 2A and 2B. Compared with the control strain, the accumulation of P3HB and P3HB content were both increased using xylose as the sole carbon source in NOG strain. The carbon yield of NOG strain was increased up to 0.24, about a 26.3% increase compared with the control strain with a carbon yield of 0.19. This result is in agreement with the computational simulation result that introducing NOG pathway could improve the P3HB yield. Considering both glucose and xylose were the most abundant sugar in lignocellulosic carbon sources. These two sugars could not be co-utilized simultaneously in *E. coli* and many other bacterial due to the carbohydrate catabolite repression (CCR). Quite different CCR metabolisms have involved in gram-negative and gram-positive organisms [9]. The xylose isomerase (encoded by *xylA*), xylulokinase (encoded by *xylB*) and arabinose transporter (encoded by *araE*) from *Bacillus subtilis* were introduced into *E.coli* to simultaneously utilize glucose and xylose due to different mechanisms of CCR between these two kind of bacteria (unpublished data). To reduce glucose repression and increase xylose assimilation, plasmid pZABE harboring genes *xylA, xylB* and *araE* from *B. subtilis* was constructed and transformed into the control and engineered strains. P3HB production and yield were also detected using 10 g/L glucose and 5 g/L xylose as the carbon source. The P3HB production was improved from 2.0 g/L to 3.0 g/L, and yield was improved from 0.21 to 0.31(Fig 2C and 2D). This result indicated that the NOG pathway was also effective for simultaneous utilization of glucose and xylose.

3 Improved PHB/glycerol yield through the introduction of NOG pathway into *E.coli*

**Figure 3.**
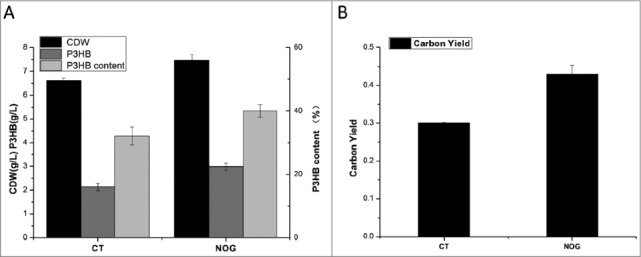
Comparision of CDW, P3HB concentration, P3HB content (A) and the carbon yield (B) on glycerol (B) in the control *E. coli* and engineered *E. coli* strains.

Experiments with shaken flasks were performed with 10 g/L glycerol as carbon source in basal medium that contained yeast extract, peptone, Na_2_HPO_4_ and MgSO_4,_, pH 7.2 [10]. The results of the cell dry weight, P3HB weight, P3HB content and carbon yield were shown in Fig 3A and 3B. The engineered strain with NOG pathway in the shake-flasks produced 2.99 g/L P3HB with the carbon yield of 0.43, which is 43.3% higher compared with the control strain. This indicates the effectiveness of NOG pathway on P3HB production using glyce rol as the sole carbon source.

## Conclusion

To reduce the production cost of PHA, the use of low cost carbon sources is urgently required. In this work, the simulation results showed that with NOG pathway introduce, both xylose-input and glycerol-input were displayed high carbon yield compared with the classical pathway. Experiments were also conducted and verified the results. Through engineering of NOG pathway into *E.coli*, the carbon yield of NOG strain was increased up to 0.24, about a 26.3% increase compared with the control strain using xylose as sole carbon source. Similar phenomenon was observed using glycerol as sole carbon source, the carbon yield of NOG strain reached 0.43, about a 43.3% increase compared with the control strain. Thus, low cost carbon sources have the potential to produce P3HB, and introducing NOG pathway into *E. coli* might be a universal strategy using xylose and glycerol as the sole carbon source.

## Abbreviations

CCR: carbohydrate catabolite repression.
FBA: flux balance analysis
NOG: non-oxidative glycolysis
P3HB: poly-(3-hydroxybutyrate)
